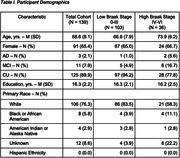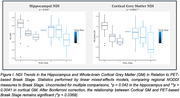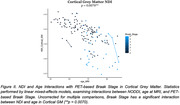# Relationships between brain microstructure and Braak Stage progression on the Alzheimer’s disease continuum

**DOI:** 10.1002/alz.095488

**Published:** 2025-01-09

**Authors:** Lauren A. Gitzlaff, Jason F Moody, Yue Ma, Sterling C. Johnson, Sanjay Asthana, Tobey J. Betthauser, Bradley T. Christian, Andrew L Alexander, Barbara B. Bendlin

**Affiliations:** ^1^ Wisconsin Alzheimer’s Disease Research Center, University of Wisconsin School of Medicine and Public Health, Madison, WI USA; ^2^ Wisconsin Alzheimer’s Disease Research Center, University of Wisconsin‐Madison, School of Medicine and Public Health, Madison, WI USA; ^3^ Waisman Center, University of Wisconsin‐Madison, Madison, WI USA

## Abstract

**Background:**

Hyperphosphorylation of tau protein underlies the accumulation of neurofibrillary tangles (NFT) in the brain, a neuropathological hallmark of Alzheimer’s Disease (AD). Braak stages, which reflect the regional spread of NFTs, are defined by NFT presence in the entorhinal cortex (Stages I and II), limbic regions including the hippocampus (III and IV), and neocortex (V and VI). Diffusion weighted imaging (DWI) is a magnetic resonance imaging (MRI) technique that sensitizes the MRI signal to the random motion of water molecules and has shown promise for detecting AD‐related microstructural brain changes, including NFT presence. Since diffusion patterns may be disrupted in NFT formation, DWI metrics may be sensitive to NFT formation. In this study, we examine the relationships between DWI metrics of neurodegeneration and Braak stage progression determined by positron emission tomography (PET) imaging.

**Method:**

139 participants from the Wisconsin ADRC and Wisconsin Registry for Alzheimer’s Prevention on the AD continuum (Table 1) were imaged with multi‐shell DWI and MK‐6240 PET within 36 months of each other. Parameter maps of Neurite Density Index (NDI), Orientation Dispersion Index (ODI) and Isotropic Volume Fraction (ISO) were computed and average values of each were extracted from the hippocampus, anterior parahippocampal gyrus, and whole‐brain cortical gray matter. We used a linear mixed‐effects model to test DWI’s ability to predict PET‐based Braak Stage, with age, sex, clinical diagnosis, DWI protocol, and the interaction between age and each metric as covariates.

**Result:**

Two significant relationships between Braak stage and NDI were found. As Braak stage increased, NDI significantly decreased in the hippocampus and whole‐brain cortical grey matter. Additionally, there was a significant interaction between cortical grey matter NDI and age. The results are uncorrected for multiple comparisons.

**Conclusion:**

Our findings suggest that, as the extent of tau accumulation increases, there is a concurrent decline in neurite density in the hippocampus and cortical grey matter, and further, the association between NDI and Braak stage progression was more pronounced in older individuals. Therefore, NDI, a metric sensitive to axonal and dendritic loss, may track AD‐related pathology. Additional work is needed to determine the specificity of DWI metrics to tangle pathology.